# The assessment of the potential hepatotoxicity of new drugs by *in vitro* metabolomics

**DOI:** 10.3389/fphar.2023.1155271

**Published:** 2023-05-05

**Authors:** Guillermo Quintás, José V. Castell, Marta Moreno-Torres

**Affiliations:** ^1^ Metabolomics and Bioanalysis, Health and Biomedicine, Leitat Technological Center, Barcelona, Spain; ^2^ Analytical Unit, Health Research Institute La Fe, Valencia, Spain; ^3^ Unidad Mixta de Hepatología Experimental, Instituto de Investigación Sanitaria del Hospital La Fe (IIS La Fe), Valencia, Spain; ^4^ Departamento de Bioquímica y Biología Molecular, Facultad de Medicina, Universidad de Valencia, Valencia, Spain; ^5^ CIBEREHD, Instituto de Salud Carlos III, Madrid, Spain

**Keywords:** metabolomics, drug hepatotoxicity, mechanisms of hepatotoxicity, *in vitro*, HepG2 cells, primary hepatocytes, biomarkers

## Abstract

Drug hepatotoxicity assessment is a relevant issue both in the course of drug development as well as in the post marketing phase. The use of human relevant *in vitro* models in combination with powerful analytical methods (metabolomic analysis) is a promising approach to anticipate, as well as to understand and investigate the effects and mechanisms of drug hepatotoxicity in man. The metabolic profile analysis of biological liver models treated with hepatotoxins, as compared to that of those treated with non-hepatotoxic compounds, provides useful information for identifying disturbed cellular metabolic reactions, pathways, and networks. This can later be used to anticipate, as well to assess, the potential hepatotoxicity of new compounds. However, the applicability of the metabolomic analysis to assess the hepatotoxicity of drugs is complex and requires careful and systematic work, precise controls, wise data preprocessing and appropriate biological interpretation to make meaningful interpretations and/or predictions of drug hepatotoxicity. This review provides an updated look at recent *in vitro* studies which used principally mass spectrometry-based metabolomics to evaluate the hepatotoxicity of drugs. It also analyzes the principal drawbacks that still limit its general applicability in safety assessment screenings. We discuss the analytical workflow, essential factors that need to be considered and suggestions to overcome these drawbacks, as well as recent advancements made in this rapidly growing field of research.

## 1 Introduction

The success rate in pharmaceutical drug development is strongly linked to an early detection of the potential undesirable effects of the new drug on humans. This has been classically examined in the early preclinical phase by testing drugs on experimental animals. Differences in the response of drugs in animals and in man, and, in particular, differences in drug metabolism, bioactivation and disposition, stimulated the search for human relevant experimental models that could replace the use of animals for human safety assessment and prediction. In line with the 3Rs policy (replacement, reduction, refinement), drug toxicity in humans is aimed to be detected across the drug discovery process without relying exclusively on animal studies. Thus, there has been a big effort in the past decades for creating new experimental methodologies that circumvent the use of animals for toxicological research, without compromising its accuracy. These non-animal strategies are faster, cheaper and provide extensive and meaningful data regarding both compound’s hazard and safety and are constantly evolving and improving. They essentially integrate human relevant cellular models and powerful analytical tools.

The use of omics approaches (particularly transcriptomics and proteomics) to assess relevant changes in cells exposed to new compounds, started many years ago, with variable success (Kennedy, 2002; Joseph, 2017). More recently, technical advances for the precise identification and quantitation of small molecules, facilitated the offspring of metabolomics and its application in drug discovery and development. This ‘omic’ has become nowadays a relevant tool for toxicology and risk assessment. Metabolomics involves the analysis of metabolites present in a biological system, and its biological interpretation. The information retrieved from the analysis of the metabolic profile permits the identification of perturbed metabolic cell reactions, pathways and networks, which are useful for the evaluation of a pathological alteration or the consequences of an external intervention, such as the exposure of cells to xenobiotics/drugs. Metabolites are low molecular weight organic molecules, usually less than 1,500 Da, covering a wide range of structural, physicochemical properties and concentration ranges. The metabolome of a cell includes different classes of molecules such as amino acids, nucleosides, fatty acids, lipids, phospholipids, sugars, steroids, organic acids and many other entities that participate in the normal cell’s metabolism (Patti et al., 2012; Johnson et al., 2016). Metabolites can serve as building blocks for larger, more complex, molecules or are the result of their catabolism. As such, they are directly involved in a vast network of biochemical processes/reactions taking place in cells. Thus, the metabolome is the direct result of the biochemical activity of a living cell and can provide a snapshot of the current status of cellular metabolism. In general, the metabolome is more closely linked to the immediate physiological state of the cell, tissue or organism, while the proteome may provide information about longer-term changes in cellular function and development (Fiehn, 2002). Metabolites are not only the downstream products of genes, mRNA and proteins, but they are also the result of other signals (epigenome), governing the performance of cells. In addition, metabolites back-regulate and affect the other “omics” layers such as transcriptomics and proteomics (Karczewski and Snyder, 2018).

The chemical space of metabolomics and its number is vast, but with advances in analytical methods that improve sensitivity and specificity, their detection is nowadays affordable. Indeed, there has been an increase in the number of metabolites that can now be easily detected and annotated. This makes it possible to identify and analyze a significant and large number of metabolites, typically in the range of hundreds, that are of great value to interpret the most relevant alterations associated with the toxic insult of a particular compound (Ramirez et al., 2013). Therefore, analyzing cell’s metabolome provides a direct view of the changes associated with the biochemical performance of a biological system (Rodríguez-Morató et al., 2018). Nevertheless, this analysis is limited to the subset of metabolites that are detected and identified in the model systems.

Metabolomics has emerged, over the last 2 decades, as a mature tool in toxicology, enabling us to obtain information about the effects of xenobiotics on target organs and improving our understanding of the modes of action of bioactive/toxic compounds. Nowadays, metabolomics is recognized as a powerful tool for assessing the positive and negative effects of drugs under different exposure conditions (Ramirez et al., 2013).

Furthermore, the combination of metabolomics with *in vitro* models represents a great step forward in the use of human-relevant non-animal alternatives in toxicology. Using human relevant *in vitro* models for hazard/risk assessment of pharmaceuticals offers several advantages over *in vivo* studies. The strategy can be applied during the early drug discovery phase, and the experimental conditions and potential confounders are more manageable, providing greater repeatability. Additionally, *in vitro* models require smaller sample sizes and offer higher throughput at a more affordable cost (Bellouard et al., 2022).

The liver plays a central role in the homeostasis of the whole organism as well as plays a key role in drug metabolism and biotransformation, with many active metabolic processes susceptible to being altered after exposure to drugs. Drug metabolism involves the chemical biotransformation of parent drugs molecules, commonly through phase I reactions (catalyzed by CYP450 and FMO enzymes) and phase II reactions (involving conjugation reactions driven by UDP-glucuronosyltransferases, sulfotransferases, N-acetyltransferases, glutathione S-transferases and methyltransferases), leading to more hydrophilic, usually inactive compounds, that are more readily excreted from the liver into urine and feces (Vaja and Rana, 2020). Notwithstanding, the results of biotransformation reactions can be harmful. The resulting metabolites can be more toxic or reactive, and these reactions can also generate intermediates that may cause damage to hepatocytes or other cells in the liver. Hepatotoxicity is typically associated with metabolic disturbances in target liver cells. Therefore, it is possible that the deleterious effects of a drug causing drug-induced liver injury (DILI) can ultimately be observed through characteristic changes in the cell’s metabolome (Moreno-Torres et al., 2022a).

Hence, one way to monitor the harmful effects of a xenobiotic on hepatocytes is by examining the intracellular metabolome. In addition, it can also be reflected in changes of the levels of extracellular metabolites (exometabolome), which, in part, reflect changes of the intracellular metabolome. Hence, it is also possible to assess the occurrence of an *in vivo* DILI episode by monitoring liver biomarkers in other biological fluids such as serum or urine, obtained by non-invasive or minimally invasive procedures, avoiding the need for liver biopsies for diagnostic purposes. Although the metabolites released from hepatocytes after drug exposure and injury are only a part of the altered endometabolome, they can still serve as potential biomarkers for hepatotoxicity *in vivo* DILI studies, as recent research has demonstrated (Quintás et al., 2021). Metabolomics *in vivo*, is an incidentally used strategy for identifying hepatic toxicity biomarkers in different biological samples such as plasma, serum, urine, feces, or tissue biopsies. However, it has the main limitation that these samples are typically collected in the clinical setting only after liver injury has already occurred and rarely at the time of the peak event. Thus, determining whether metabolic changes observed *in vivo* are the either the cause or the result of a toxicity event, represents a significant challenge in metabolomics (Luo et al., 2021).

On the other hand, *in vitro* metabolomics can overcome the limitations of *in vivo* studies and reveal early events following drug exposure. This can lead to a better understanding of the sequence of initiating molecular events that lead to the toxic effect, and ultimately to the description of an adverse outcome pathway (AOP) that outlines the sequence of molecular and cellular events of toxicity. Thus, *in vitro* metabolomics is a valuable tool for screening hepatotoxicity both *in vitro* and *in vivo*. However, due to its inherent complexity, *in vitro* metabolomics presents drawbacks that can limit its general applicability in safety assessment screenings.

In this review, we aim to provide an up-to-date overview of recent *in vitro* hepatotoxicity studies that made use of metabolomics to assess the potential hepatotoxicity of drugs. We have reviewed the analytical workflow and the key factors that need to be improved to overcome current limitations. We also discuss the progress that has been made in terms of methods and practices in this emerging area of research.

## 2 Metabolomics *in vitro*: the experimental design for robustness and data reproducibility

As mentioned before, metabolomics facilitates the understanding of the biochemical phenomena occurring in cells or tissues exposed to a potentially toxic compound. For this, it is crucial to have a properly study design, the right experimental model, careful sample collection and processing, and appropriate data analysis in order to accurately interpret the biological significance of the results. Additionally, standardized procedures and internal controls must be used to ensure reproducibility of data.

**FIGURE 1:**
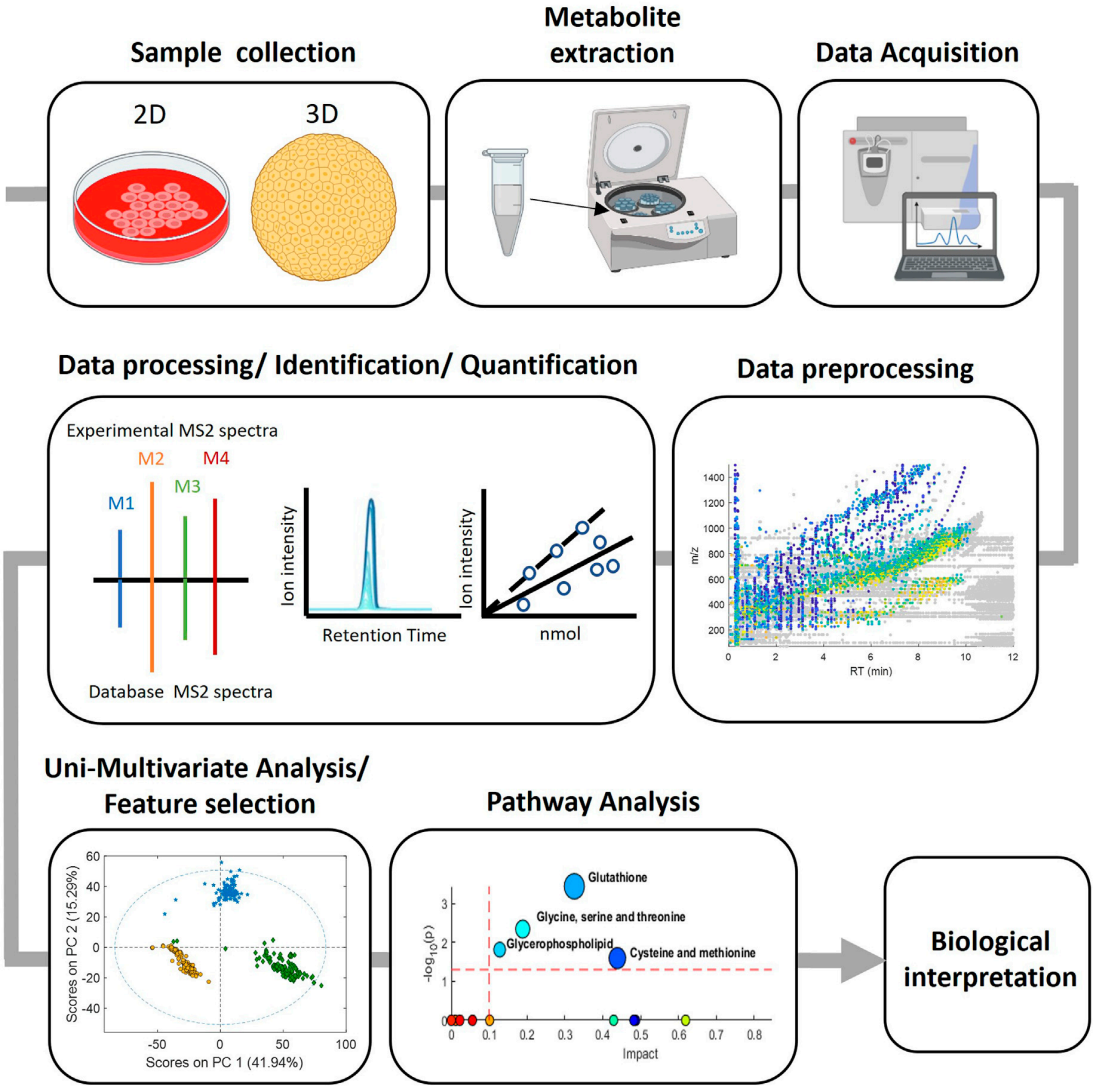
Experimental workflow in metabolomic analysis.

The whole strategy involves a series of sequential steps, that are schematised in Figure 1 and typically involves the following steps: 1) Sample collection, where biological samples are collected from the study subjects according to the experimental design, 2) Metabolite extraction, where depending on the nature of the sample and the metabolites of interest different approaches such as solvent extraction, protein precipitation and solid-phase extraction can be used, 3) Data acquisition, various analytical techniques such as GC or LC-MS are used to detect and quantify the metabolites present in the prepared samples, 4) Data preprocessing, the generated data is pre-processed for chromatographic peak detection, alignment and normalization procedures to remove sampling differences, batch effects or injection order-dependent signal drifts, 5) Data processing with bioinformatic tools for the identification and quantification of the detected metabolites and 6) Biological interpretation which involves statistical analysis to identify differences between groups and correlate metabolites with biological processes or clinical outcomes as well as to provide insights into metabolic pathways, biomarker discovery and disease mechanisms. Key to this strategy is to ensure a proper identification of metabolites that are representative of the cell’s targets, signaling pathways, and biochemical pathways, relevant for toxicity evaluation.

### 2.1 Experimental models for *in vitro* hepatotoxicity assessment

The results and significance of metabolomic analysis done *in vitro* are largely dependent on the characteristics of the different hepatic cell types used and their specific requirements.

Experimental *in vitro* models comprise a wide range of cell types and culture procedures, including primary hepatocyte cultures, differentiated hepatic cell lines, stem cell derived hepatocytes, cultured on two- (2D) or three-dimensional (3D) formats, which can significantly influence the outcome of the experiment. Toxicology assessments through *in vitro* metabolomic analysis require striking a balance between using liver cell models with appropriate metabolic capabilities, while ensuring their ease of handling and reproducibility of data. Hepatic cell types can exhibit significant variations in their metabolic performance (Kim et al., 2014; Cuykx et al., 2018), which can be further influenced by various culture conditions, such as cell passage, senescence level, and differentiation status (Moreno-Torres et al., 2021). These factors should be considered when designing metabolomics studies to ensure the reliability of the results.

Despite the potential of *in vitro* metabolomics to assess liver toxicity, its use is still infrequent, as indicated by the scarcity of research papers on this subject. Several *in vitro* models have been used for investigating the hepatic metabolism of drugs and xenobiotics and their potential hepatotoxic effects by means of metabolomic analysis. Primary Human Hepatocytes (PHHs) are considered the gold standard of vitro models for predicting *in vivo* metabolism because they can mimic hepatic metabolic reactions, including those catalyzed by cytochrome P450 (CYP) enzymes and by flavin-containing monooxygenases (FMOs), as well as conjugation with glucuronic acid, sulfate, and glutathione to a large extent. Among the 29 studies reviewed, only one utilized PHHs as the model system (Xu et al., 2020). Although PHHs serve as a valuable *in vitro* model for many applications, they have several important limitations. One of the most significant issues is their high inter-donor variability, which significantly impairs data reproducibility and limits further bioinformatic data analysis. Additionally, their in-time availability might be problematic, they have a short lifespan and their metabolic performance decays *in vitro* over time.

Immortalized hepatoma cell lines, such as L-02, derived from normal embryonic liver cells and Huh-7 (a well differentiated cell line derived from a human carcinoma) have also been used. HepaRG (a cell line isolated from a cholangiocarcinoma, that can be differentiated *in vitro* to adult hepatic cells) and HepG2 (a differentiated cell line exhibiting epithelial-like morphology, isolated from a hepatocellular carcinoma) are widely available and have been used extensively in liver metabolism and hepatotoxicity studies. L-02 cells are a cost-effective alternative to PHHs for initial hepatotoxicity screening in humans. Among the studies reviewed, L-02 cells have been frequently utilized to study hepatotoxicity mechanisms due to their excellent proliferative capacity and ability to perform typical liver cell functions (Cuykx et al., 2019a; Hu et al., 2019; Zhao et al., 2019; Dong et al., 2020; Liu et al., 2020; Wang et al., 2020; Zhang et al., 2020; Zhang et al., 2021).

HepaRG cell line is the second most used cellular model and is generally considered as a good surrogate for PHHs when investigating liver metabolism and detoxification (Rodrigues et al., 2018; Cuykx et al., 2019a; Cuykx et al., 2019b; Seeger et al., 2019; Manier et al., 2020; Bellouard et al., 2022; Iturrospe et al., 2022). It presents low inter-batch variability and long-term stability while expressing most liver-specific functions including CYP activity and bile acid synthesis (Cuykx et al., 2018). HepaRG cells are dually capable of being differentiated towards hepatocyte-like cells and to biliary-like cells, mimicking the *in vivo* situation (Guillouzo et al., 2007; Marion et al., 2010; Iturrospe et al., 2022). Furthermore, HepaRG cells have also proven to be valuable for *in vitro* cholestatic research (Anthérieu et al., 2012; Woolbright et al., 2016; Rodrigues et al., 2018). However, previous results indicate that the number of significantly altered metabolites in HepaRG exposed to α-pyrrolidinobutiophenone (α-PBP) and α-pyrrolidinoheptaphenone (α-PEP) is lower that in human liver microsomes (HLM) or PHHs. This outcome could be attributed to the capability of HepaRG cells to differentiate into hepatocyte-like cells that exhibit significant levels of CYP3A4 expression, while their expression of CYP2D6 is comparatively lower (Manier et al., 2020).

Despite HepG2 and Huh-7 display low biotransformation capacities, they both have been used [Huh-7 (Liu et al., 2019; Krajnc et al., 2020; Krause et al., 2021) and HepG2 (Chatterjee et al., 2018; Luo et al., 2021; Moreno-Torres et al., 2021; Martínez-Sena et al., 2023)] as *in vitro* models for hepatic toxicity metabolomic studies, because of their stability. When testing drug-induced hepatotoxicity *in vitro*, 2D cell cultures are currently the most commonly used culture system because of its simplicity in handling and culture requirements. They are also easy to process, quench and extract for further metabolomic analysis. However, the 2D monolayer cell cultures do not fully reflect the features of hepatocytes *in vivo*. This limitation influences the cell’s response to drugs, potentially altering the drug’s mechanisms of action, or boosting the cell’s resistance to the drug (Bonnans et al., 2014). Additionally, some hepatotoxicity key events rely on interactions between cells, a feature that is not well-displayed in 2D cultures.

Spheroids, a 3D cell culture technique that closely mimics the cell status/environment within the liver, can produce results that more accurately reflect the *in vivo* changes in metabolic pathways in response to toxic compounds, as compared to 2D cell culture (Silva et al., 2013). A 3D culture model can mimic more accurately the microenvironment in which cells proliferate, aggregate, and differentiate, leading to improved predictions of drug hepatotoxicity on a cell, in particular, in repeated doses or long-term toxicity. This is due to the model’s ability to resemble more closely the organotypic histomorphology and microenvironment of hepatocytes in the liver *in vivo* (Lv et al., 2017), the impact of metabolic cooperation between different cell types on metabolic profiling and long-term survival being metabolically competent. As a result, 3D culture models are becoming increasingly popular (Tung et al., 2011) and reduce, at the same time, the need for animal testing in toxicity evaluations (Tutty et al., 2022). We found that about 20% of the metabolomic-based hepatotoxicity studies reviewed used 3D models involving either L-02 cell spheroids (Silva et al., 2013) or HLMs (Wang et al., 2019; Goracci et al., 2020; Zhou et al., 2020; Kim et al., 2021). Although 3D culture models offer several advantages, such as improved physiological relevance, they present greater difficulties in terms of their handling due to variations in size, cell number, and cell composition. Additionally, the minute amounts of metabolites produced by spheroids pose a challenge for analytical tools, often pushing them to the limits of their performance.

Research has shown that using 3D spheroid cultures of L-02 cells to investigate the hepatotoxicity of perfluorooctanoic acid (PFOA) through non-targeted metabolomics can provide valuable insights into the chronic toxicity mechanisms of PFOA, that cannot be uncovered in 2D monolayer models (Silva et al., 2013).

HLMs are capable of reactions catalyzed by membrane-bound enzymes such as CYP enzymes, FMOs, and uridine 5′-diphospho-glucuronosyltransferases (UGTs) which are among the most relevant ones in drug metabolism. However, while HLMs provide one of the most convenient ways to assess CYP-mediated metabolism they hardly predict *in vivo* metabolism of phase II metabolism other than glucuronidation, or the combination of both phase I and II metabolism (Manier et al., 2020). These 3D microtissues (MTs) biological matrices were used in an innovative combination with a lipidomics approach and a cheminformatic workflow for data analysis, aiming at anticipating drug effects end points in an early discovery phase, mimicking subchronic exposure conditions (Goracci et al., 2020). In a previous study, MTs had already been characterized and validated for predicting DILI which demonstrated to be superior to cytotoxicity results in conventional 2D PHHs cell cultures (Proctor et al., 2017).

To summarize, 2D cell models of cell lines are the easiest to handle and most reproducible models for *in vitro* toxico-metabolomic studies. However, they may not fully capture all aspects of a specific type of hepatotoxicity, such as drug-induced cholestasis. In such cases, 3D models may be more appropriate, but researchers must be prepared to handle the increased complexity and variability of these models during experimentation.

### 2.2 Experimental factors: concentration and exposure times

When cells are exposed to a chemical, multiple metabolic changes may occur. These specific changes will depend on the chemical and biochemical effects (e.g., mode of action) of the chemical, as well as on the concentration and the exposure time. It is therefore important to determine for each cell system the appropriate drug concentration and exposure time, in order to assess the type of metabolic impact and the mechanism of toxicity. The drug concentration used in the study should exceed the lowest observed effect level (LOAEL) *in vitro*, but not cause significant cell death. The objective is to select a concentration that triggers the primary molecular events leading to toxicity, without surpassing a threshold that produces metabolomic signals linked to overall cytotoxicity and significant cell death. Thus, it is recommended to perform a dose toxicity curve prior to conducting the studies, and it is advised that the concentration chosen for metabolomic hepatotoxicity assessment should not reach IC50 level. In the studies reviewed, a range of concentrations were usually tested, and cytotoxicity was prior assessed using different endpoints, such as MTT (Rodrigues et al., 2018; Hu et al., 2019; Zhao et al., 2019; Liu et al., 2020; Zhang et al., 2020; Bellouard et al., 2022; Martínez-Sena et al., 2023), neutral red uptake (Cuykx et al., 2019a; Cuykx et al., 2019b; Iturrospe et al., 2022), CCK8 assay (Liu et al., 2020; Xu et al., 2020; Luo et al., 2021), CytoTOX-ONE homogeneous membrane integrity assay and WST-1 cell proliferation assay (Seeger et al., 2019; Krause et al., 2021), Real Time-Glo Annexin V Apoptosis, Necrosis reagent (Krajnc et al., 2020) or by measuring the propidium iodide red fluorescence levels (Silva et al., 2013). The concentration/toxicity curves acquired from cytotoxic assays are also utilized to determine the point of departure (PoD), which is the concentration leading to significant metabolic alterations in the *in vitro* systems. Choosing a drug concentration for metabolomics studies based on cell alteration end-points instead of cell death outcomes is crucial. This approach allows for the early identification of metabolic pathway changes associated with the primary molecular events that lead to liver injury. By doing so, the recorded events are specifically induced by the substance, rather than being a result of overall cytotoxicity and cell death (Krajnc et al., 2020). In fact, inhibitory concentrations such as IC10 or fractions of these values are often preferred over IC50 for this purpose (Rodrigues et al., 2018; Bellouard et al., 2022; Iturrospe et al., 2022).

When selecting the exposure concentration, it is also important to consider other physiological factors, such as the route of administration and distribution, and pharmacological factors, such as Cmax, to prevent the use of unrealistic exposure scenarios (Cuykx et al., 2019b). Lower, rather than high concentrations are chosen to mimic real levels found in blood and liver from treated-patients (Bellouard et al., 2022). Xu et al. observed that N, N-dimethylformamide (DMF) treatment significantly released ALT to culture media in a dose-dependent manner and claimed that ALT might serve as a sensitive biomarker for DMF-induced liver injury, compared to cell viability that was only affected at the highest concentrations (Xu et al., 2020). Supplementary Table S1 summarizes the concentration ranges analyzed in the reviewed results.

When assessing multiple drugs to compare their potency in altering the cellular metabolome and intrinsic differences, it is recommended to incubate them at the same concentration. This concentration should not result in significant cell death in cultured hepatocytes, regardless of the clinical Cmax differences of the drugs (Goracci et al., 2020).

The toxic event, which progresses from molecular initiating events to secondary events and ultimately results in severe cell dysfunction and death, can be best described as a sequential process occurring over time. So, apart from the effects that are dependent on the dosage, there are also cellular metabolic alterations that depend on time during the course of a toxic event. Therefore, the duration of exposure is a crucial factor in *in vitro* metabolomics toxicity studies. Thus, the timing usually selected to uncover such molecular initiating events by means of metabolome alterations typically ranges between 30 min and 24 h. Longer incubation times can make it challenging to identify the early metabolic impairments directly caused by the exposure to the drug. Nevertheless, longer-term exposures are frequently studied, often in combination with multiple time points, to examine the time-course variation of the metabolome, to understand the sequence of molecular initiating events leading to an adverse outcome pathway, and chronic toxicity upon repeated exposure to a given compound (Silva et al., 2013; Rodrigues et al., 2018; Cuykx et al., 2019b; Goracci et al., 2020).

### 2.3 Importance of including control and negative reference compounds

A key aspect in drug-toxicity evaluation by *in vitro* metabolomics is the importance of including data from both non-hepatotoxic compounds as well as non-treated cells (Cuykx et al., 2019b). Precautions should be taken to minimize confounding signals that could be attributed to the experimental conditions rather than to the substance being tested, given the dynamic nature of the metabolome and its susceptibility to alteration by small external stimuli. Culture conditions *in vitro* metabolomics studies, for instance, are often overlooked but can be a significant source of bias in the data, sometimes even more intense than the effects of the drug being tested. Even the presence of a non-toxic bioactive xenobiotic can cause metabolic alterations that need to be distinguished from those caused by a hepatotoxic substance. Without careful consideration and monitoring of these confounding factors, the data obtained can be misleading in interpreting the toxicity events. Comparing the metabolome of cells exposed to a hepatotoxin and to a non-toxic negative control can provide a first insight into the toxic mode of action. However, due to the limited scope of small-scale experiments (e.g., one concentration, one time point exposure), this is not always done, leading to great uncertainties on the predictive value of the observed metabolic alterations. Cuykx et al. evaluated the metabolomic impact of sodium saccharin which is considered a non-hepatotoxic chemical and thus a suitable compound to identify markers of xenobiotic exposure, not related to hepatotoxicity. They observed that several lipids (n = 15) changed significantly, including increased levels of triacylglycerols and decreasing levels of phospholipids in the culture media (Cuykx et al., 2019b). However, they identified common alterations between non-hepatotoxic reference compounds used as negative control and hepatotoxic compounds, which demonstrate the importance of these controls to eliminate background and reduce false positive results when investigating toxicological insults.

Martínez-Sena et al. (2023), incorporated larger numbers of non-hepatotoxic compounds (citrate, ketotifen, 3-aminophenol, ascorbic acid, betaine, dexamethasone, gentamicin, glucose, lactose and N-acetylcysteine) in their experimental design. Interestingly, metabolic alterations were detected with all these non-hepatotoxic compounds, but the pattern of changes differed from that of hepatotoxic compounds (Martínez-Sena et al., 2023).

Another aspect to consider *in vitro* metabolomic analysis is the occurrence of hyperosmolarity which is a consequence of a too high concentration of the xenobiotic being assayed. This phenomenon has been described for human cell cultures as leading to altered levels of intracellular monosaccharides and amino acids which allows the cell to equilibrate the osmotic balance (Cuykx et al., 2019b). To prevent biased selection of metabolites altered by a compound’s mode of action, it is important to intentionally avoid hyperosmolar concentrations. This is especially relevant *in vitro* experiments, where concentrations can be easily increased beyond therapeutic levels.

## 3 Sample preparation and metabolomic analysis

The optimization of cell processing to immediately block metabolism and ensure proper extraction of cell metabolites for further analysis, is essential to ensure robust, reproducible and meaningful metabolomic data.

### 3.1 Quenching and extracting metabolites

Speeding up the process of extracting metabolites from cells by quickly processing samples and halting or quenching metabolic activity is critical. Commonly used quenching methods in metabolomics studies include washing the culture plates and adding pre-cooled methanol or acetonitrile, or rapidly freezing the plates in liquid nitrogen. To minimize the contamination of the endometabolome by the exometabolome during the extraction process, it is crucial to perform quenching rapidly and with care to avoid metabolite leakage. Once quenched, the cells are typically transferred to eppendorf tubes for further processing.

It is often recommended to include a prior washing step, such as rinsing culture plates with cold PBS immediately before quenching, to minimize carryover effects. Introducing a washing step when using adherent 2D cultures is simpler since it can be done very quickly before quenching. However, the impact of the washes on metabolite leakage in suspended cells or spheroids that require a centrifugation step must be thoroughly examined. Adding a washing step in cell suspension or 3D cultures also comes with potential drawbacks such as the unavoidable larger quenching time frame (Kapoore and Vaidyanathan, 2016).

To extract metabolites, various factors need to be considered, such as the physicochemical properties of metabolites, including their solubility in polar and nonpolar solvents, and their molecular size.

Standard operating procedures with optimized extraction processes including internal standards for quality control of the process are critical and should be rigorously applied to all samples to obtain reproducible results. Solvent mixtures containing methanol, acetonitrile and/or chloroform, with or without water, are commonly used for metabolite extraction in the *in vitro* toxicometabolomic studies reviewed. Biphasic solvent systems, such as methanol/chloroform/water, offer several advantages over single-phase solvent systems. By extracting both polar and non-polar metabolites in a single sample, this method can reduce variability caused by analyzing them separately. Following centrifugation, both fractions can be analyzed separately providing a better metabolomic coverage (Kapoore and Vaidyanathan, 2016). However, optimized methods tailored to the specific type of matrix and target metabolites may be necessary to achieve the optimal results in targeted analysis (Lin et al., 2007; Chen et al., 2013; Medina et al., 2020; Yan et al., 2020).

### 3.2 Sample analysis

In metabolomics studies of hepatotoxicity, there are two main strategies: targeted and untargeted. Targeted approaches focus on measuring and quantifying specific metabolites, while untargeted approaches aim to gather as much metabolic information as possible. In the case of mass spectrometry (MS)- based metabolomics, several strategies are use, e.g., desorption electrospray ionization mass spectrometry (DESI-MS) or flow injection analysis-MS (FIA-MS), or in combination with separation techniques such as gas chromatography-mass spectrometry (GC-MS), liquid chromatography-mass spectrometry (LC-MS), or capillary electrophoresis-MS (CE-MS), to increase separation and better identification and quantitation of the metabolites present in a cell extract (Pitt, 2009; Moros et al., 2017; Seeger et al., 2019; Dong et al., 2020).

In order to maximize the effectiveness of *in vitro* metabolomic studies for hepatotoxicity, it is important to have a comprehensive coverage of the cell’s metabolome. However, due to practical limitations, a balance must be achieved between comprehensiveness and feasibility. A common approach involved the combination of complementary analytical methods or separation techniques aiming at specific metabolic classes (e.g., lipids, amino acids).

Cuykx et al. employed a combination of reversed phase (C19 column) and hydrophilic interaction liquid chromatography (HILIC) chromatography for a better coverage of the metabolome in the non-polar and polar fractions, respectively (Cuykx et al., 2019a; Cuykx et al., 2019b). Liu et al. (Liu et al., 2020) evaluated the sensitivity and reproducibility of an HILIC method, assessed the influence of mobile phase gradient, ionic strength and column temperature and validated the method for glycolysis pathway metabolites upon exposing Huh-7 cells to 2,3,7,8-tetrachlorodibenzo-p-dioxin (TCDD) (Goracci et al., 2020). Dong et al., used also a combination of complementary analytical methods including gas chromatography with flame ionization detection (GC-FID) and MS (GC-MS) for fatty acid analysis, LC-MS/MS for ceramide and sphingosine analysis and 1H-NMR to retrieve a global metabolic profile (Dong et al., 2020). Iturrospe et al. used a headspace GC-FID to quantify ethanol in cell media and a Drive-Tube Ion-Mobility (DTIM)-QToF-MS for metabolomic and lipidomic analysis (Iturrospe et al., 2022). Rodrigues et al. used an LC-MS/MS approach for the determination of cholic acid and glycocholic acid and 1H NMR for global metabolomic analysis of cell culture media (Rodrigues et al., 2018).

### 3.3 Metabolite annotation

In order to interpret untargeted LC-MS data in a biological context, it is necessary to annotate the detected features using computational tools. However, the percentage of LC-MS features annotated is typically lower than 20%. This limitation hampers biochemical interpretation and identification of toxic events in metabolomics studies.

**FIGURE 2:**
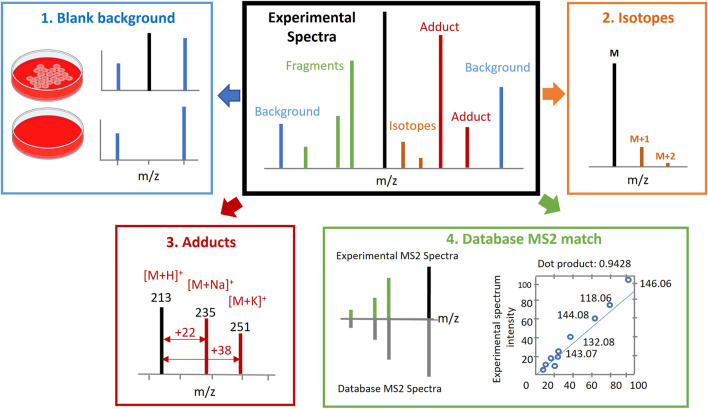
Metabolite annotation. Several considerations should be accounted for besides accurate mass (AM) and retention time (RT). This includes elimination of background signals (Kennedy, 2002), deisotoping (Joseph, 2017), recognition of adduct peaks in spectra (Patti et al., 2012) and mass spectrometry/mass spectrometry (MS/MS) fragmentation pattern that is compared to in house or publicly available databases or internal standards, and the matching degree is estimated (Johnson et al., 2016). Figure adapted from (Kim and Kang, 2021).

This involves the comparison of mass fragmentation spectra (MS/MS spectra) experimentally acquired in the study through data dependent acquisition (DDA) or data independent acquisition (DIA) against the spectral and retention time (RT) databases of known or predicted metabolites. Several considerations are typically accounted for, besides accurate mass and RT, which includes the elimination of background signals, deisotoping, identification of adduct peaks in MS spectra, and MS/MS fragmentation patterns compared to databases or internal standards and the matching degree estimated (see Figure 2).

Metabolite annotation in toxico-metabolomic studies has been carried out using different mass spectra databases, including HMDB (Rodrigues et al., 2018; Cuykx et al., 2019b; Hu et al., 2019; Wang et al., 2019; Krajnc et al., 2020; Liu et al., 2020; Xu et al., 2020; Zhang et al., 2020; Moreno-Torres et al., 2021; Bellouard et al., 2022; Feng et al., 2022; Martínez-Sena et al., 2023), METLIN (Cuykx et al., 2019a; Cuykx et al., 2019b; Manier et al., 2020; Luo et al., 2021; Moreno-Torres et al., 2021; Bellouard et al., 2022; Feng et al., 2022; Iturrospe et al., 2022; Martínez-Sena et al., 2023), mzCloud (Silva et al., 2013; Hu et al., 2019; Zhao et al., 2019; Xu et al., 2020; Zhang et al., 2020; Zhang et al., 2021), KEGG (Hu et al., 2019; Zhao et al., 2019; Xu et al., 2020; Zhang et al., 2020), NIST (Manier et al., 2020; Iturrospe et al., 2022), GNPS (Iturrospe et al., 2022), LipidMaps or Chemspider (Cuykx et al., 2019a; Cuykx et al., 2019b), Metware database (Liu et al., 2020), LipidMatch, LipidHunter, MS-Finder, MassBank (Iturrospe et al., 2022) or by comparing them with an in-house library build through the analysis of available standards (Goracci et al., 2020; Bellouard et al., 2022).

While community guidelines for metabolite identification have already been published, their adoption has been limited due to the diversity of LC-MS data acquisition methods and the use of non-standardized manual curation workflows. Hence, there is a general lack of a standardized reporting procedure for MS/MS spectra acquisition and metabolite annotation. Furthermore, it is often the case that published results do not include detailed information on the quality score for the match between experimental and database peaks, the fragmentation conditions used, or the quality thresholds used for the experimental spectra (Manier et al., 2020).

To assist with the reporting of annotation confidence levels, it may be helpful to make raw data available for sharing through platforms such as Metabolights and the Metabolomics Workbench. Automated processing and easy-to-report parameters can also help to streamline the annotation process. Several considerations should be accounted for, besides accurate mass (AM) and retention time (RT), which includes the elimination of background signals, deisotoping, recognition of adduct peaks in spectra, and MS/MS fragmentation pattern compared to databases or internal standards and the matching degree estimated (Figure 2).

### 3.4 Profiling the exo- and endo-metabolome

In some situations, analyzing the exometabolome (i.e., the metabolites released by released cells), can be advantageous because it allows direct assessment of the metabolome changes in the culture media without disrupting the cell culture or extracting the metabolites from the cellular matrix, simplifying the monitoring of time-course events. While changes in the endometabolome, or intracellular metabolites, may be reflected to some extent in the exometabolome, this is not always the case. However, exometabolome analysis can be very useful for assessing drug metabolism and metabolic effects linked to toxic events, provided that these metabolites are released from the cells and remain stable in the culture media until sample collection and preprocessing.

Sample processing and methods for analyzing the endometabolome and exometabolome differ. One major challenge is the high concentration of nutritional components present in culture media, such as amino acids, glucose, nucleosides, and vitamins that can hinder the detection of metabolic changes linked to the hepatotoxic events that can be up to several orders of magnitude lower.

Several studies analyzed the exo-metabolome in culture media to examine hepatotoxic effects of xenobiotics (Rodrigues et al., 2018; Seeger et al., 2019; Wang et al., 2019; Goracci et al., 2020; Manier et al., 2020; Xu et al., 2020; Yadav et al., 2020; Kim et al., 2021; Iturrospe et al., 2022). Goracci et al. analyzed the culture media supernatant to identify dronedarone, entacapone and metformin metabolites using liver micro tissues competent in both Phase I and II xenobiotic metabolism (Goracci et al., 2020). Other studies performed media metabolite profiling for the study of drug metabolism using incubations with recombinant CYPs or HLMs (Wang et al., 2019; Yadav et al., 2020; Kim et al., 2021). Iturrospe et al. used metabolomic data from cell culture media to quantify the concentration of a hepatotoxic agent, in their case ethanol, pre and post cellular incubations, to confirm the incorporation of the xenobiotic into the cells (Iturrospe et al., 2022). Manier et al. compared the alterations observed in the endometabolome and exometabolome for the identification of biomarkers of hepatotoxicity of the two new psychoactive substances α-PBP and α-PEP, and concluded that the features found in cell culture media in these experiments had better discriminant properties and were more suitable biomarkers than the significant features found within cells (Manier et al., 2020). Other studies also compared the endometabolome and exometabolome (Seeger et al., 2019) by metabolomics and lipidomics to assess the occurrence of hepatotoxicity, with variable discriminating power (Rodrigues et al., 2018; Xu et al., 2020).

The analysis of the exometabolome is a valuable tool for evaluating drug-induced liver damage, particularly in cases where obtaining a liver sample from DILI patients is not feasible for medical and ethical reasons. The exometabolome results from *in vitro* studies can provide insights into the metabolites that may be also present in the serum or plasma of DILI patients. However, it is crucial to ensure that the intracellular metabolites of interest are released from hepatocytes during the course of an hepatotoxic insult and remain stable in the blood, in order to be used as reliable biomarkers for the diagnosis and monitoring of DILI. Therefore, the potential clinical application of exometabolome findings is evident and warrants further investigation (Moreno-Torres et al., 2022a).

### 3.5 Main sources of variability in *in vitro* testing

**FIGURE 3:**
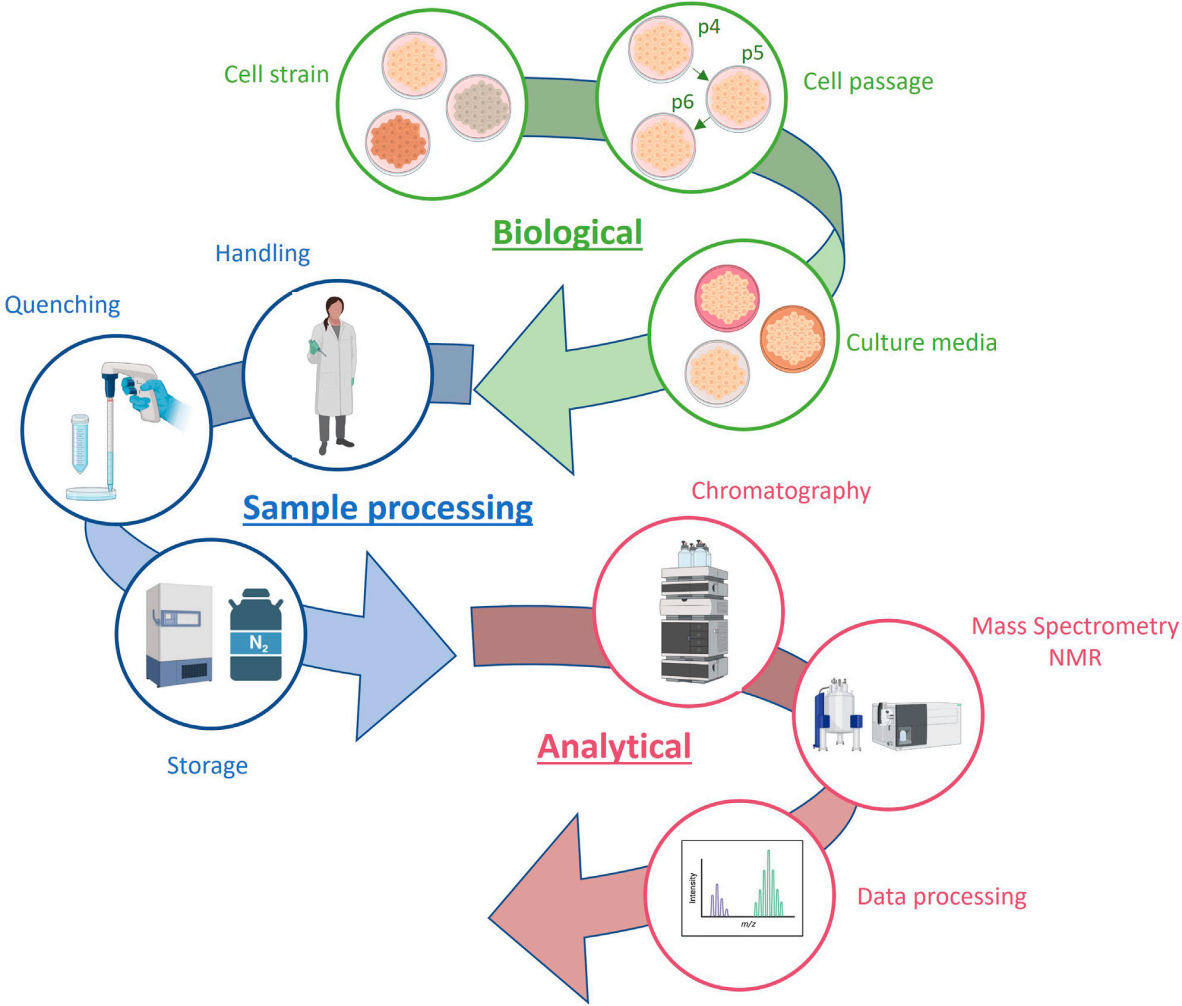
Sources of variability in metabolomics data from *in vitro* hepatotoxicity studies.

Obtaining reliable results in *in vitro* toxicology studies requires a comprehensive understanding of the biological system and the inclusion of controls for all potential influencing factors, such as cellular passage and processing batch (Moreno-Torres et al., 2021). These studies often involve analyzing numerous samples under various experimental conditions, presenting a significant challenge in controlling the variability resulting from different drugs, concentrations, incubation times, and analytical batches. The main sources of variability in metabolomics data from *in vitro* hepatotoxicity studies are summarized in Figure 3.

A key issue, common to all cellular systems, is the variability introduced with the use of cultures of different cell passage numbers. Failure to account for this, adds variability, reduces the reproducibility and the power of downstream statistical analysis. Moreno-Torres et al. examined the effects of cell passage, sample processing batch, and instrumental batch drifting on metabolomic profiles of a series of identical samples, to examine how these factors interact (Moreno-Torres et al., 2021). Results obtained showed that all these factors influence on data reproducibility, with sample processing batch and storage time being the most disruptive.

Sources of variation related to sample manipulation can be minimized and partially controlled using well defined standard operational procedures (SOPs). SOPs are lists of concise, step-by-step written instructions, that document all the procedures followed for data acquisition. SOPs are fundamental to maintain quality control (QC) and quality assessment (QA) processes and facilitate reproducible research within and across laboratories. Although not frequently adopted in academic research laboratories, one cannot sufficiently emphasize the importance of defining and having a strict attrition to SOPs, even in academic metabolomic studies. For example, using the same type of collection tubes and processing methods for all samples to minimize variability, the use quality control samples to detect and normalize any variability, to store samples at a constant and appropriate temperature and avoid temperature fluctuations, to prevent exposure to moisture and oxygen which can lead to oxidation and degradation of certain metabolites and to standardize quenching procedures and temperature for all samples, are strongly recommended. Furthermore, there are several types of quality control samples that are typically used in metabolomics analysis to ensure accuracy and reliability of metabolomics data and helping to identify any sources of variability or bias in the analytical process (Broadhurst et al., 2018). In general, biological variations resulting from media preparation, inoculum densities or pre-cultivation factors are significantly higher than the analytical or instrumental variance. To measure this variation, it is recommended to conduct experiments with a sufficient number of biological replicates. Studies reviewed have described a range of three to eighteen replicates, with six being the most common number used.

The robustness of an *in vitro* metabolomics test system alone is not enough to ensure the reliability and reproducibility of the data obtained. Gradual variations in the LC-MS instrumental response within and between batches can lead to unwanted and uncontrolled data variation, ultimately reducing the repeatability and reproducibility of the analysis, and difficulting the extraction of biological information.

**FIGURE 4:**
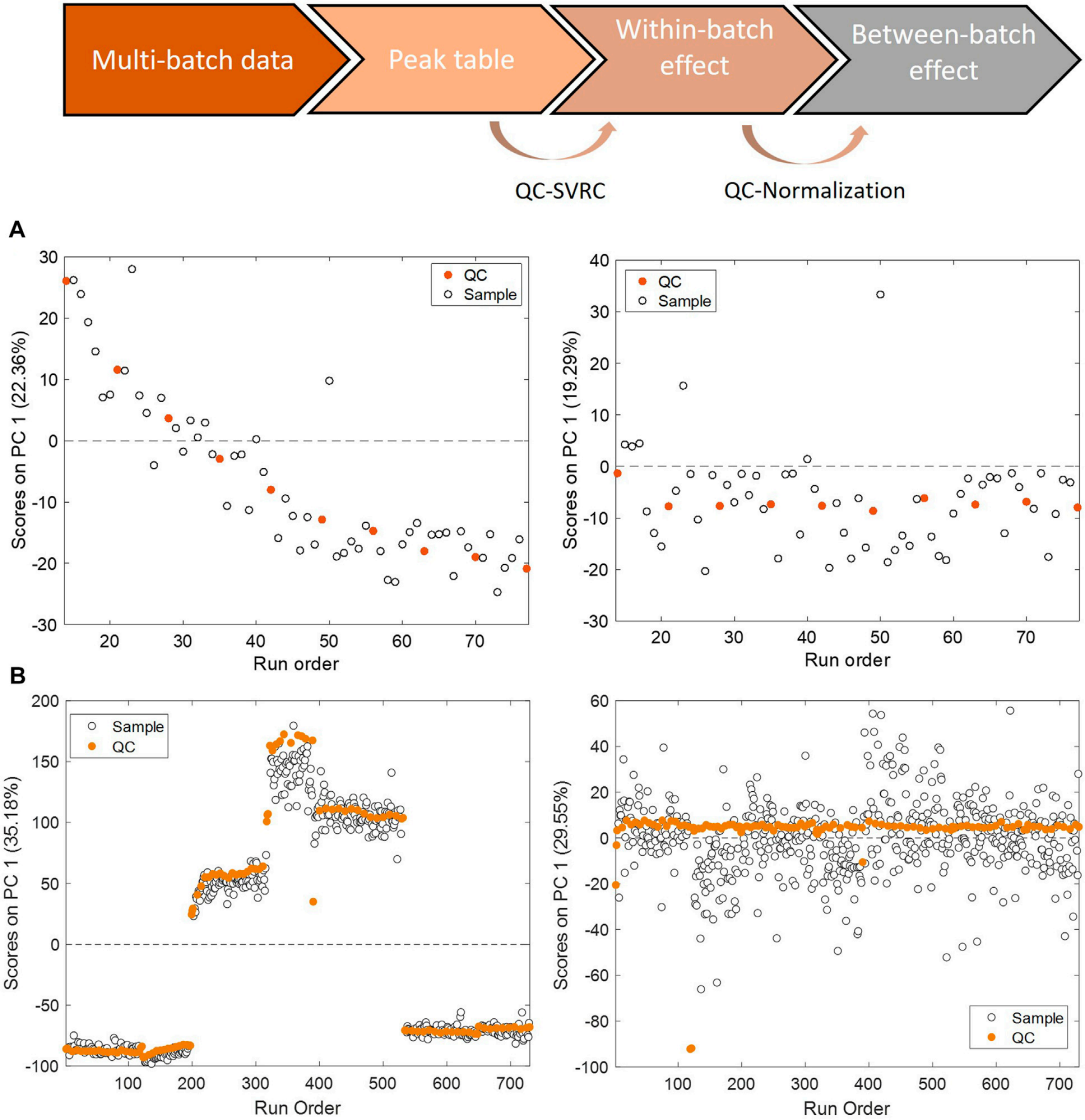
Within and between batch effect correction using QC samples. PCA scores as a function of the injection order calculated for QCs in the example data set before (left) and after (right) QC-SVRC correction **(A)** for within batch effect elimination or QC normalization **(B)** for inter batch effect elimination.

Changes in instrumental conditions such as inlet interface contamination, ionization efficiency, mobile phase composition or column performance, introduce a systematic bias in the instrumental response, and can also reduce its precision. In this situation, the signal retrieved for a given analyte is not only dependent on the concentration in the sample, but also on the relative position of the sample in the batch analysis. This so-called within-batch effect, decreases the repeatability and reproducibility as well as the discriminant power to detect relevant biological responses, and difficults data interpretation and reuse. Batch effects are often unavoidable and thus, a very active field of research. To overcome this challenge, it is important to apply post-acquisition chemometric techniques for batch correction to achieve reliable high-throughput screening (Kuligowski et al., 2014; Kuligowski et al., 2015). A common procedure involves the use of pooled QC samples, distributed throughout the analytical batch. The response obtained from the analysis of the QC is used for the monitoring of the instrument stability (Silva et al., 2013; Cuykx et al., 2019a; Cuykx et al., 2019b; Zhao et al., 2019; Liu et al., 2020; Zhang et al., 2020; Bellouard et al., 2022; Feng et al., 2022; Iturrospe et al., 2022). Furthermore, the analytical variation in the response observed in pooled QC samples can be used to model the instrumental drift and thereafter to correct the within-batch effects. Principal component analysis (PCA) can be used to conduct an unsupervised assessment of the batch effect in toxico-metabolomic studies. This method generates new variables, referred to as Principal Components (PCs), which effectively capture the most important sources of variation in the data. By summarizing these patterns, PCA enables a visual representation of the structural characteristics in the data. The PCA scores as a function of the injection order is shown in Figure 4. The scores of the first PC accounting for 22% and 35% of the total variance showed significant associations with the injection order and the batch origin. These two sources of variation are evidenced in Panel A and B, that illustrate trends in PCA scores as a function of the injection order corresponding to within-batch effects, and Panel B, where a tight clustering among batches indicative of a between-batch effect respectively. On Panel A, the PCA of data set before and after (left and right panels) within-batch effect correction using support vector regressions (QC-SVRC) is displayed. On Panel B the PCA of the dataset before and after (left and right panels) QC correction for between batch effects is represented. After QC normalization the observed within and between batch clustering effects were no longer detected, indicating a successful removal of both biases.

The elimination of batch effects is critical for reusing publicly available data sets and for the merging of data acquired in different time points, batches, instruments or laboratories.

Several linear (Manier et al., 2020) and non-linear strategies (QC-Robust Smoothing Splines (QC-RSC) (Kirwan et al., 2013), QC-Support vector regression correction (QC-SVRC)) (Kuligowski et al., 2015; Moreno-Torres et al., 2021) have been proposed for the modeling of within-batch effects.

Information retrieved from the analysis of QCs can also be used to identify unreliable features showing RSD% or MSD% higher than a user-selected threshold (typically in the 10%–20% range).

Goracci et al. identified the batch effect as one of the most critical issues in metabolomic applications when used for screening purposes (Goracci et al., 2020). Goracci et al. conducted a study that mimicked real-world conditions by spreading the drug treatments and analyses over a 6-month period, divided into four batches. This approach not only involved variation in UHPLC-MS instrumental performance but also included human liver MTs produced in different batches from the same cell lots. Principal Component Analysis (PCA) allowed the identification of the batch as the main source of variation. To correct for this batch effect, they performed an automatic merging of lipid profiles from replicates, followed by subtraction of the lipid profile of each control of the corresponding treated sample, on a given day of treatment.

Despite the importance of the use of QC in untargeted metabolomics, few authors make use of this correction (Moreno-Torres et al., 2021; Iturrospe et al., 2022; Martínez-Sena et al., 2023). Nine reviewed publications did not report the utilization of QC samples for data normalization (Silva et al., 2013; Cuykx et al., 2019a; Cuykx et al., 2019b; Hu et al., 2019; Zhao et al., 2019; Luo et al., 2021; Zhang et al., 2021; Bellouard et al., 2022; Feng et al., 2022) and even 16 publications did not include QCs in their analysis (see Supplementary Table S2). Therefore, standardizing protocols for QA/QC in toxico-metabolomics is crucial for improving the quality and reproducibility of data in this type of studies, and to facilitate the joint analysis and re-use of data from different studies and sources.

### 3.6 Data normalization

Data normalization helps minimize the impact of non-biological factors on metabolite concentration and is essential in any toxico-metabolomic study.

Normalization is crucial for accurately analyzing and comparing *in vitro* metabolomics data as it corrects for errors and slight variations caused by factors such as changes in the number of living cells, cell harvesting, sample processing, and detection sensitivity. There are two general strategies for normalization. Pre-acquisition normalization, involves normalizing the extracted metabolome to a metric that is expected to have an even spurious influence on all of the metabolite signals retrieved from a sample using, for example, the cell number. Finally, it is important to ensure that the chosen cell sample is an accurate representation of the entire cell population. However, during the process of scraping or trypsinization, some cells are inevitably lost, leading to changes in the metabolic pattern and reducing the accuracy of normalization (Wu and Li, 2016). Additionally, cells within the sample may have differing physiological states, and therefore may not precisely reflect the metabolic status of the whole population (Kapoore et al., 2015). Alternative normalization factors employed include the diameter of spheroids in 3D models (Goracci et al., 2020), total DNA content, image cell counting of culture plate attached cells by micrograph digitalization or normalization to dry cell weight (DCW) (Seeger et al., 2019) and total protein content (Zhang et al., 2022). Normalization to DCW can be problematic because it is a time-consuming process, requires a large number of samples, and it can introduce a significant amount of weighing errors. Normalization to protein content has shown poor correlation to cell number in some cases (Silva et al., 2013). Both approaches require separate samples which might introduce bias and reduce the accuracy of the normalization.

On the other hand, post-acquisition options for normalization are based on the retrieved metabolic profiles. These include the use of the peak area normalized to the internal standards (Wang et al., 2019; Manier et al., 2020), the use of the total area of the spectrum (Chatterjee et al., 2018; Xu et al., 2020; Luo et al., 2021), a probabilistic quotient normalization (Cuykx et al., 2018) by the median of the QC pooled samples (Iturrospe et al., 2022), or by the MS total useful signal from the NOREVA online software (Bellouard et al., 2022).

Despite the impact that normalization may have in correcting the noise from external variating factors, most reviewed results did not include a normalization step in their data processing pipeline (Cuykx et al., 2019b; Hu et al., 2019; Krajnc et al., 2020; Liu et al., 2020; Wang et al., 2020; Yadav et al., 2020; Zhang et al., 2020; Zhou et al., 2020; Kim et al., 2021; Krause et al., 2021; Zhang et al., 2021; Feng et al., 2022).

## 4 Identification of biomarkers and metabolic pathways in drug hepatotoxicity assessment

**FIGURE 5:**
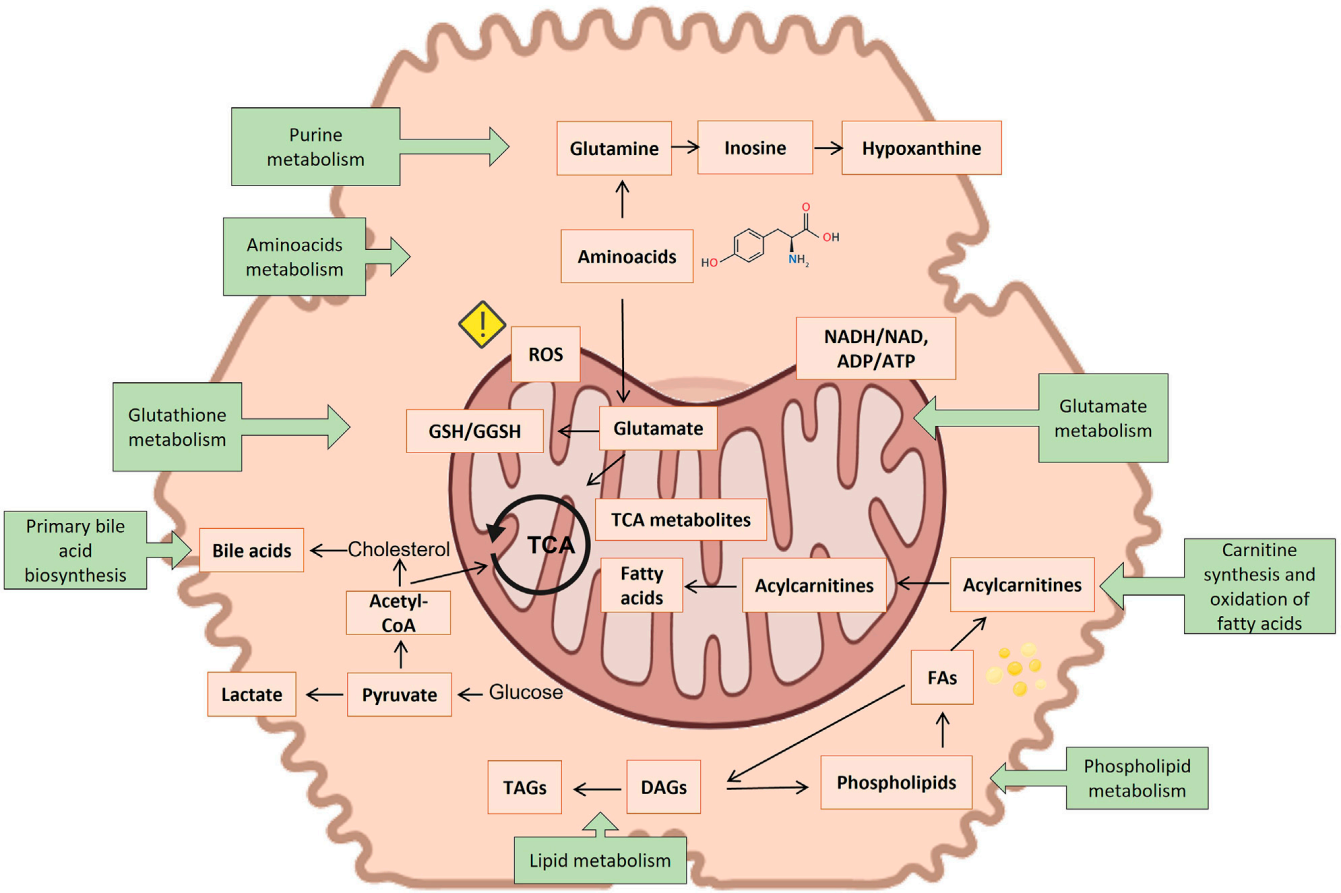
Suggested connection between different identified metabolite markers of toxicity (orange squares) and the most reported metabolic pathways of toxicity (green squares). Created with BioRender.com.

Several metabolites emerge as biomarkers of hepatocyte injury from metabolomic studies on hepatotoxicity and are gathered in Supplementary Table S3. It contains a compilation of hepatotoxic compounds assayed, metabolites altered, and the altered metabolic pathways so far involved. Figure 5 summarizes the observations of the toxicity papers reviewed with the most commonly perturbed metabolites and metabolic pathways.

Of all metabolites so far reported, carnitine and acylcarnitines were consistently the most repeatedly identified biomarkers in the course of many hepatotoxic events. The tendency reported in the different studies is not always consistent, increasing in some studies, while decreasing in others. Carnitine, a N-methylated γ amino acid, is responsible for transporting fatty acids across the inner mitochondrial membrane for beta-oxidation. There is a link between plasma levels of carnitine and mitochondrial dysfunction (Adeva-Andany et al., 2017) and acylated carnitines have been related to steatogenic processes (Meissen et al., 2015; Olsvik et al., 2017). Although carnitine appears as the most frequently altered metabolite, the metabolic pathway of carnitine synthesis appeared significantly altered, only in two investigations (Xu et al., 2020; Zhang et al., 2020).

The second most commonly altered metabolite is glutathione, either in the reduced or in the oxidized state and the metabolic pathway of glutathione (GSH) metabolism appeared altered in 8 studies. GSH homeostasis is largely regulated in the liver and plays a major role as an antioxidant and cell-signaling regulator (Ten-Doménech et al., 2022). It has been found to be an essential element for detoxifying free radicals and reactive oxygen species (ROS), thereby serving as the primary defense against oxidative stress induced by cellular respiration in the mitochondria of cells (Ten-Doménech et al., 2022). It also plays a crucial role in defending the cell against drug-derived radical or reactive oxygen species emerging in the course of an hepatotoxicity event (Güntherberg and Rost, 1966). A decreased level of GSH makes hepatocytes more vulnerable to oxidative irreversible damage in the course of hepatotoxicity. However, although a decreased GSH/GSSG ratio has been defined as a hallmark for oxidative stress, it is not exclusive or specific to hepatotoxicity. Despite its high interest, GSH and GSSG titration is challenged by the pre-analytical enzymatic- and non-enzymatic GSH oxidation (Yuan and Kaplowitz, 2009). A commonly used approach to prevent the oxidation of GSH makes use of the Michael addition reaction for the alkylation of the thiol group of GSH with N-ethylmaleimide (NEM) (Heidari et al., 2015). Nonetheless, pre-analytical derivatization of the thiol group is not used in the majority of the metabolomic studies reviewed. Nonetheless, pre-analytical derivatization of the thiol group is not used in the majority of the metabolomic studies reviewed. Ophthalmic acid (L-γ-glutamyl-L-α-aminobutyrylglycine), a tripeptide analog of glutathione in which the cysteine group is replaced by L-2-aminobutyrate, is a good indicator of oxidative stress, and is also altered in several of the reviewed studies (Xu et al., 2020; Martínez-Sena et al., 2023).

Oxidative stress is also a downstream effect of other toxicological molecular initiating events, for example, uncoupling of the tricarboxylic acid cycle (TCA). Indeed it also appears to be associated with altered levels of malic acid, succinic acid and citric acid (Hu et al., 2019; Zhao et al., 2019; Liu et al., 2020; Xu et al., 2020; Zhang et al., 2020; Luo et al., 2021; Moreno-Torres et al., 2021).

Additionally, the disruption of electron flow in the respiratory chain due to an impaired mitochondrial respiration also leads to a reduced reoxidation of NADH into NAD+, thereby reducing oxidation of pyruvate by the pyruvate dehydrogenase complex. This consists of several enzymes. Collectively they transform pyruvate, NAD+ and coenzyme A into acetyl-CoA, CO2, and NADH. The conversion is crucial because acetyl-CoA then may be used in the citric acid cycle to further fostering cellular respiration. The inability to consume pyruvate alternatively results in its reduction to lactate, causing lactic acidosis (Rodrigues et al., 2018; Hu et al., 2019; Zhao et al., 2019; Dong et al., 2020; Wang et al., 2020; Zhang et al., 2020; Luo et al., 2021; Moreno-Torres et al., 2022a; Feng et al., 2022). The higher concentrations of acetate and lactate often recorded suggest an elevated rate of glycolysis to counteract an impaired fatty acid oxidation in the mitochondria, as a major source of energy (ATP) in hepatocytes. The increase of acetoacetate levels, once again, denotes a decrease in mitochondrial function (Begriche et al., 2011; Chatterjee et al., 2018; Rodrigues et al., 2018; Luo et al., 2021).

Other biomarkers frequently associated with hepatotoxicity events, although not fully specific, are choline and phosphatidylcholines. Choline and its derivatives have many functions, the most notable being a precursor for phospholipids as well of signaling molecules. Phosphatidylcholines are a structurally important component of cell membrane and essential for their stability. They also participate in the biosynthesis of S-adenosylmethionine. Phosphatidylcholines are needed for the synthesis of VLDLs. Choline promotes fat transportation and improves the utilization of fatty acids, preventing an abnormal accumulation of fat in the liver (steatosis). A lack of choline leads to the accumulation of lipids in the liver (Hensley et al., 2000). As a therapeutic drug it is widely used for treating non-alcoholic fatty liver disease, as well other liver disorders (Borges Haubert et al., 2015). Choline had a preventive effect on oxidative damage in mice (Li et al., 2016). Choline deficiency has been found to be associated with the onset of liver cancer in rats (Inoue et al., 2007). Lower levels of choline may result in steatosis induced by certain medications. In that sense phosphocholine appeared frequently altered in hepatotoxicity studies. Several of the studies reviewed reported changes in different phospholipids, and although no clear mechanism is established linking phospholipids changes to DILI, mitochondrial dysfunction, oxidative damage, or inflammation have been suggested. It is also possible that excessive accumulation of phospholipids in tissues, especially in hepatocytes, may be responsible for liver toxicity *via* vacuolization of hepatocytes and necrosis (Iturrospe et al., 2022).

Liver damage can also lead to modifications in amino acid metabolism, which is usually displayed as a decrease in the levels of branched-chain amino acids and an increase in aromatic amino acids. The decreased levels of leucine/isoleucine and valine observed in several studies (Rodrigues et al., 2018; Zhao et al., 2019; Liu et al., 2020; Feng et al., 2022), suggest their abnormal catabolism, which implies amino acid imbalance.

Elevated levels of other amino acids have been also reported (Chatterjee et al., 2018; Liu et al., 2019; Zhao et al., 2019; Wang et al., 2020; Xu et al., 2020; Zhang et al., 2020; Luo et al., 2021; Bellouard et al., 2022; Feng et al., 2022; Iturrospe et al., 2022; Martínez-Sena et al., 2023) including arginine, lysine, phenylalanine, histidine, tryptophan, methionine and alanine. Hepatocyte injury and dysfunction interrupts active protein biosynthesis in the liver and lack of their utilization, leading to increased levels of free amino acids in both the liver and blood. The changes in the intracellular concentrations of amino acids differed depending on the severity of liver damage (Moreno-Torres et al., 2022a).

L-Glutamate is another amino acid that is commonly altered in metabolomic hepatotoxicity studies. It reacts with ammonia, catalyzed by glutamine synthetase to form non-toxic glutamine, helping to decrease blood ammonia levels and preventing ammonia encephalopathy (Bai et al., 2013). Research also indicates that L-glutamate is able to prevent fatty liver in a cholesterol-fed rabbit model (Yanni et al., 2010). Furthermore, glutamine is involved in the biosynthesis of antioxidants such as GSH. Indeed, it has been shown that supplementation with glutamine increases the intracellular levels of GSH in the 3D L-02 spheroids (Zhang et al., 2022).

Besides analyzing changes in individual metabolites, as biomarkers of hepatotoxicity, the effects of any kind of stressor on model organisms can be also evaluated at a higher integration level by determining their influence on metabolic pathways. A metabolic pathway is classically described as a series of linearly connected chemical reactions that feed one another. The pathway starts with one or more metabolites and, through a series of intermediates, converts them into products. The metabolic pathway analysis aims at identifying clusters of metabolites related to key cellular events and metabolic networks and provides mechanistic insight into the underlying biochemistry of differentially expressed metabolites. However, metabolic pathways are not simple one-way roads, rather they are part of a complex network of interactions. Based on this view, each molecule becomes a node in this and in other networks. When cells are exposed to toxicants, either these nodes may change (altered metabolite concentration) or remain relatively constant, or the connections originating from them may change (altered metabolic flux). Since metabolic pathways are interconnected, the perturbation of a single metabolite can affect more than one metabolic pathway. By interpreting the perturbations occurring upon drug treatment at the metabolic pathways level, a more comprehensive understanding of the impact of drugs on the complexity of cell’s metabolic networks can be achieved. Thus, it can help identifying which metabolic pathways are most deeply involved and perturbed in the course of an hepatotoxic event, and provide valuable insight into the mechanisms and initiating molecular events of the toxic event (Chong et al., 2019).

**FIGURE 6:**
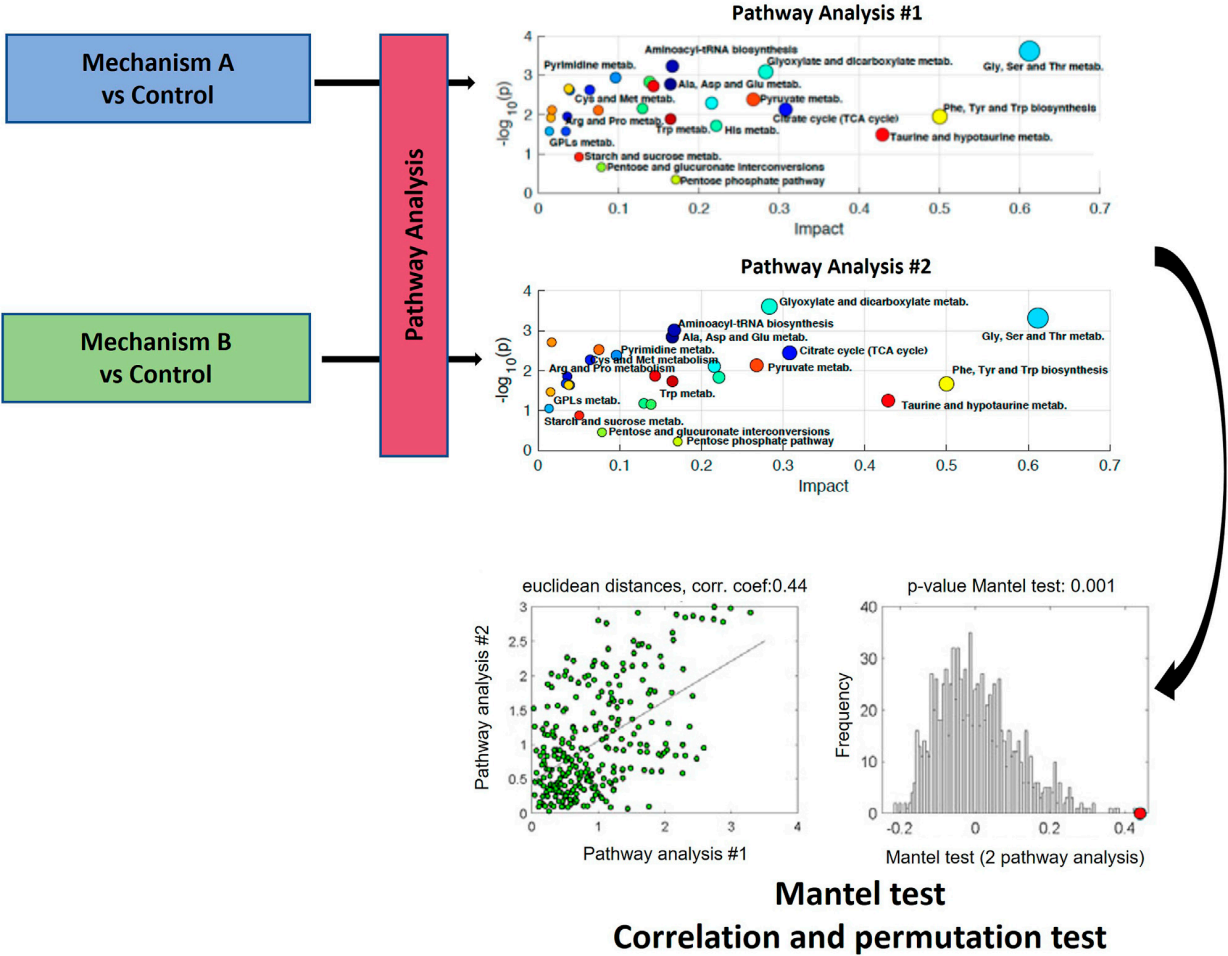
Schematic workflow for the functional correlation analysis of results from metabolic pathway analysis. The Mantel’s test is based on the estimation of the statistical significance of the correlation between two matrices summarizing the results from pathway analysis. Figure adapted from (Liu et al., 2015; Moreno-Torres et al., 2021; Martínez-Sena et al., 2023).

**FIGURE 7:**
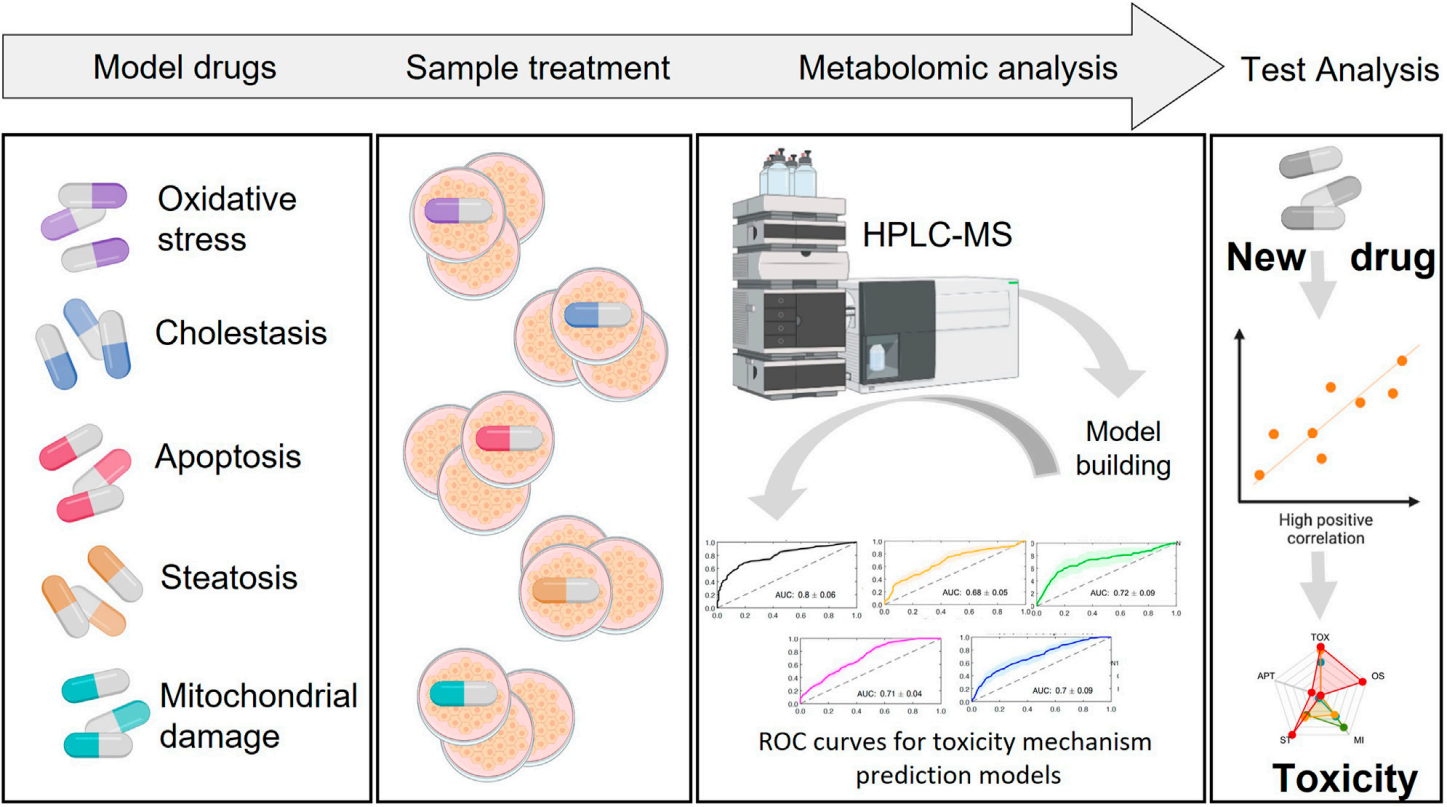
Building prediction models. Incubation of cells with a set of known hepatotoxic compounds for the generation of a metabolic fingerprint for different toxicity mechanisms. Based on this, build predictive models that enable to evaluate the likeliness of a novel/unknown compound to be hepatotoxic and through which mechanism.

Moreno-Torres et al. and Martínez-Sena et al., evaluated whether the analysis of the impact on a set of metabolic pathways could constitute a characteristic metabolic fingerprint of the different hepatotoxicity mechanisms (Moreno-Torres et al., 2021; Martínez-Sena et al., 2023). This view constitutes a further step in the in-depth data analysis of metabolomics pathway analysis approaches. The proposed method involves aligning the descriptors from two distinct pathway analysis, resulting in two data matrices, X1 (m x k) and X2 (m x k), which contain the descriptors for the m pathways present. The use of a correlation-based analysis of results from metabolic pathway analysis is proposed to compare pairs of multidimensional pathway information matrices. In their studies, output results from pathway analysis, which are the -log10 (*p*-value), and the impact factor (estimated as the sum of the importance measures of all metabolites in the pathway) were used as k descriptors (i.e., coordinates) of the metabolic pathway outcome (Kapoore and Vaidyanathan, 2016; Moreno-Torres et al., 2021) (Figure 6). Both works utilized Mantel test on the correlation of functional results from metabolic pathway analysis (Figure 7). The Mantel test is based on the estimation of the correlation between two matrices summarizing the results from pathway analysis. First, the dissimilarity of the two original data matrices is calculated. Then, the correlation between the two vectors built after unfolding the upper triangular parts of the dissimilarity matrices is calculated, and its statistical significance is estimated by permutation testing. This involves randomly permuting the elements in one of the two vectors and calculating the correlation coefficient for each permutation. This process is repeated multiple times to create an empirical null distribution of the set of correlation coefficients. The one-tailed (right) *p*-value, which represents the empirical estimate of the statistical significance of the correlation coefficient, is determined by calculating the proportion of permutations for which the correlation coefficient value is greater than or equal to the original statistic derived from non-permuted data as previously described (Kapoore and Vaidyanathan, 2016; Moreno-Torres et al., 2021). This correlation analysis among pathway analysis descriptors suggested that, despite some metabolic pathways are commonly altered across mechanisms, the joint analysis of a set of pathways improves the identification of toxicity mechanisms.

This approach has already been applied to the examination of reproducibility in *in vitro* metabolomics studies (Moreno-Torres et al., 2021), for the analysis of the generalization of the outcomes from metabolomic studies, for the comparison of metabolic phenotypes, and for the evaluation of the degree of differentiation of hepatocyte-like cells (Moreno-Torres et al., 2022b), which demonstrates its applicability and power as an additional source of information. Together, these applications demonstrate the algorithm’s suitability for interpreting data and conducting meta-analyses, thereby promoting its use in metabolomic research.

Drug reactive metabolites are thought to be a major trigger of hepatotoxicity, in particular of idiosyncratic metabolic DILI. Reactive intermediates are linked to reported hepatic side effects, but the occurrence of reactive metabolites does not necessarily result in hepatotoxicity, unless the defense systems of hepatocytes are overwhelmed. Nevertheless, the formation of reactive metabolites is a warning signal for potential hepatotoxicity and deserves to be examined to understand their mechanistic role in hepatotoxicity (Kim and Kang, 2021).

In this context, metabolomics has demonstrated its utility for profiling drug metabolism and identifying potential reactive intermediates (Li et al., 2011; Liu et al., 2015; Xie et al., 2018; Lu et al., 2020; Ten-Doménech et al., 2021).

For example, Yadav et al. performed a metabolism study with bromfenac to scrutinize the formation of reactive intermediates and their linkage to the hepatotoxicity of this compound (Yadav et al., 2020). *In vitro* studies have revealed that bromfenac requires conjugation by UGT’s to form a bromfenac glucuronide intermediate that cyclizes to a major metabolite, bromfenac indolinone (BI). The results demonstrated that CYP2C9 was majorly responsible for the formation of hydroxylated bromfenac and the subsequent bioactivation to thioether adducts when incubated with GSH. CYP1A2 and CYP3A4 were responsible for the bioactivation of BI by forming hydroxylated metabolites on the aromatic ring of the indolinone moiety. The aromatic hydroxylated BI is a precursor to the quinone methide and quinone imine reactive toxic intermediates in the proposed bioactivation pathway.

The integration of ‘omics’ data (e.g., transcriptomic, proteomic and metabolomic) into the risk assessment of chemical mixtures has great potential to reveal combined effects at a mechanistic level, providing insights into modes of action or adverse outcome pathways. Omic approaches used in toxicology provide a tool to characterize and quantify the molecular and biochemical changes in cells, tissues and organisms following exposure to chemicals and toxic substances. These approaches measure effects across a range of biological pathways simultaneously and can be used to investigate a chemical’s mode of action, predict toxicological effects, characterize dose response relationships, and understand species relevance. Although single-omics analyses have led to the identification of biomarkers for certain types of toxicants and exposures, they cannot provide a systemic understanding of toxicity pathways or adverse outcome pathways. Integration of multiple omics data sets offer a substantial aid to improve our knowledge on the pathway response to a toxicant. The Organization for Economic Cooperation and Development (OECD), nowadays promotes and encourages the combined use of “omics” to evaluate chemical safety.

## 5 Predictive models

Predictive classification models are one of the usable outcomes of metabolomics. They can assist in identifying metabolites and pathways that are associated with drug-induced toxicity and assist in developing most suitable drugs. An ultimate goal in the field of toxicometabolomics, and in particular drug hepatotoxicity is the use of metabolite patterns to predict the toxicological effects of unknown chemicals in the liver. To do so, metabolite patterns associated with well-known training compounds are used to develop classification schemes. Once established and validated, these schemes can then be applied to analyze the metabolite patterns of unknown compounds and based on this to predict their potential toxicity. This approach has the potential to warn researchers to identify potential hazards before drugs are tested in animals or humans (Ramirez et al., 2013). The generation of a statistical model for toxicity endpoint prediction itself represented a valuable tool for fast screening in drug discovery. Furthermore, the models presented could be improved by adding new compounds in the training set when new data are available.

The general workflow for the establishment of prediction models involves the following steps. First, a training set of known compounds belonging to different well-identified mechanisms of toxicity are evaluated in cells. Upon cell incubation, the cell extracts are analyzed by MS techniques and metabolomic data is retrieved, metabolites annotated and metabolomic patterns associated to each specific mechanism of drug-induced toxicity are identified. For model generation, a suitable machine learning algorithm is selected and the data is split into training and testing set. The metabolome of a subset of treated cells with compounds belonging to the same mechanisms of toxicity are compared to the metabolome of a subset of control treated cells (either non-toxic compounds or vehicle treated cells). Subsequently, metabolite changes and metabolic pathways altered are identified for each toxicity mechanism and predictive models, based on these identified relevant biomarkers, are built for each individual mechanism of toxicity. Usually, multivariate discriminant algorithms (e.g., PLS-DA, support vector machine, random forest) are optimized and evaluated using internal figures of merit estimated by cross validation (CV) such as the area under the receiver operating characteristic curve (AUROC), sensitivity, specificity or accuracy to assess its discriminant performance. Afterwards, the statistical significance of the model can be evaluated by permutation testing to assess the lack of overfit. Once the model is trained, a validation set generated using test compounds not included in the training set can be applied to obtain external figures of merit. The application of these models in toxico-metabolomic research can help identify potential drug toxicity issues early in drug development process, allowing for timely intervention (Figure 7). It is important to note that the use of predictive classification models in metabolomic drug toxicity studies is still in juvenile stages and requires further research and validation to assess its robustness and performance.

Goracci et al. built a model with the lipid fingerprint for hepatotoxicity which was robust and sensitive to dose treatment (Goracci et al., 2020). For that purpose, a data set of 22 drugs belonging to five different therapeutic and chemical classes with various DILI effects were selected. These included glitazones (insulin sensitizing agents), leukotriene D4 receptor antagonists (LTRA), inhibitors of catechol-O-methyl-transferase (COMT), biguanides, benzofuran derivatives and endothelin receptor antagonists. They disclosed a lipidomic profile that matched, within pharmaceutical classes, the different DILI rank categories. Preliminary tests confirmed the prediction capability of the model for new drugs and its drug-dose relationship. Regarding the hepatotoxicity potential across therapeutic drug classes, authors demonstrated that their approach was highly informative as compared to traditional assays and indicative of new mechanistic hypotheses. Furthermore they observed significant variations in the lipid fingerprint over time, and that certain drugs belonging to the same class looked similar in the grade of lipid variation but the direction in the PCA space was different, which reinforces the idea that multiple time points are recommended to be analyzed in order to understand the hepatotoxic profile and evaluate the predictions, which are limited in single time point studies (Goracci et al., 2020).

Martínez-Sena et al. (2023) assessed the metabolic alterations that arise when cells are exposed to both hepatotoxic and non-hepatotoxic compounds. They also investigated how different mechanisms of toxicity contribute to the overall hepatotoxicity of a novel compound. Selection of a training set of compounds acting through different hepatotoxicity mechanisms, was made on the basis of solid bibliographic references of scientific literature, as well as on their own expertise. Thus, 29 chemicals were chosen for which there was a clear consensus about their mode of action and preferential mechanism of hepatotoxicity, classifying them accordingly into five major mechanisms groups: oxidative stress (OS), mitochondrial disruption (MI), apoptosis (APT), steatosis (ST) and cholestasis (CHOL).

The validation set consisted of 69 hepatotoxic and 18 non-hepatotoxic compounds. The researchers evaluated the global hepatotoxicity and the presence of any of the aforementioned mechanisms of toxicity at various concentrations. They assessed the global alterations caused by a drug (i.e., toxicity index) presence and the degree of participation of any of the aforementioned mechanisms of hepatotoxicity. The most relevant metabolites for global hepatotoxicity assessment and for each mechanism prediction model (VIP>1.5) were guanosine, cysteine, N-Acryloylglycine, pyroglutamic acid, glutamylcysteine, carnitine, choline, glycerophosphocholine, N-Methylglutamic acid, ophthalmic acid, AMP, propionylcarnitine, histidyl proline, succinyladenosine, betaine, glutamyl-threonine, N-lactoyl-glycine, N8-acetylspermidine, N1, N12,-diacetylspermine, N-acetyl-L-histidine, pantothenic acid, lysoPC (16:0), N-acetylglutamic acid, 3-methylindole.

The analysis also yielded comparative data within a group of compounds, which enabled them to rank them based on their level of toxicity. They conclude that the toxic effects of a given drug may result in a set of metabolic changes which are shared by more than one mechanism, and it is interpreted as evidence of the involvement of more than one toxic pathway. This is something that was observed in several of the molecules studied, where the contribution of other mechanisms to global hepatotoxicity evolved with increasing concentrations of the compound.

## 6 Future perspectives

Metabolomics is to become an important tool in research related to the investigation of drug-induced hepatotoxicity, allowing researchers to assess the deleterious effects of xenobiotics by identifying changes in metabolite levels that uncover the toxic mechanisms at play, the identification of biomarkers of interest suitable for clinical diagnosis and facilitating the development of predictive models.

In recent years, there has been an increase in the use of metabolomics in the study of the toxic effects of pharmaceuticals *in vitro* settings. These studies have led to significant progress in the application of omics sciences in toxicology. The technical advances experienced in the field of mass detection have made MS based metabolomics a reliable, highly sensitive and versatile tool. Nevertheless, further research is needed to reduce the impact of existing technical limitations that hinder reproducibility, standardization, data comparison across laboratories, as well as compatibility with *in vitro* models. A must, if we expect to integrate this technical approach in clinics, drug development or regulatory toxicity assessment processes. QA and QC procedures are critical aspects for the use of toxico-metabolomics from a regulatory perspective. Fostering the application of QA procedures to ensure that testing is conducted consistently, accurately and following established QC procedures to increase the accuracy and reliability of the outcomes, will facilitate the application of toxico-metabolomic strategies in drug development areas.

An additional bottleneck in untargeted metabolomics is metabolite annotation. In the recent past years, this has greatly improved thanks to advancements in MS and computational metabolomic strategies.

We would like also to pinpoint the importance that in addition to metabolomic studies, in -depth studies on biochemical pathways using a multi-omics approaches in *in vitro* hepatotoxicity studies, such as transcriptomics, proteomics, and metabolomics, provides a more comprehensive understanding of the effects of xenobiotics at cellular level. By simultaneously analyzing the levels of multiple types of biomolecules, such as genes, proteins, and metabolites, it will be possible to gain insights into the complex interactions between these pharmacological compounds and the underlying biological processes that are affected. Thus, integration of the results from different omics technologies will be key to enhance functional interpretation of the data and also requires intensive data processing.

Altogether, metabolomics is currently demonstrating its value in *in vitro* drug hepatotoxicity studies, providing mechanistic information of hepatotoxic events arised upon exposure of hepatic cells to xenobiotics. Despite current limitations highlighted in this review, implementing appropriate controls and recommendations from the metabolomics community will make this field move forward improving the overall quality and comparability of the data which is required for drug screening purposes. Although the focus of this paper is based on the study of the metabolomics potential for hepatotoxicity evaluation across different drug classes, the workflow and the methods described here are easily translatable to other fields of research. This review does not only provide valuable insights into the application of metabolomics in hepatotoxicity evaluation but also offers a flexible and adaptable framework that can be employed in other research areas where metabolomics approaches could be useful or necessary. By demonstrating the versatility and reproducibility of the methods outlined in this revision, we hope to encourage further exploration and application of metabolomics in diverse fields of scientific inquiry.

## Figures and Tables

**Table:** 

**AcCa**	Acyl-carnitine
**ALT**	Alanine aminotransferase
**AM**	Accurate Mass
**ANOVA**	Analysis of Variance
**AOP**	Adverse outcome pathway
**APAP**	Acetaminophen
**API**	Apinegin
**APT**	Apoptosis
**ATP**	Adenosine triphosphate
**BCAAs**	Branched chain amino acids
**BHT**	Butylhydroxytoluene
**cAMP**	Cyclic adenosine monophosphate
**CE**	Capillary electrophoresis
**Chol**	Cholestasis
**CKK8**	Cell counting kit 8
**CMAP**	Camellia vietnamese active peptides
**CMP**	Cytidine monophosphate
**CoA**	Conezyme A
**COMT**	Catecol-O-methyl transferase
**CTD**	Cantharidin
**CYP**	Cytochrome P450 monooxygenases
**DCW**	Dry cell weight
**DESI**	Disruption electrospray ionization
**DG**	Diacylglycerol
**DILI**	Drug-induced liver injury
**DMF**	N-dimethylformamide
**DTIM**	Drive-Tube Ion-Mobility
**ERAs**	Endothelin receptor antagonists
**ESI**	Electrospray ionization
**FAD**	Flavin adenine dinucleotide
**FIA**	Flow injection analysis
**FID**	Flame ionization detection
**FS**	Fisetin
**FMN**	Flavin mononucleotide
**FMOs**	Flavin-containing monooxygenases
**GC**	Gas chromatography
**GDP**	Guanosine diphosphate
**GSH**	Glutathione
**GSSG**	Glutathione disulfide
**HILIC**	Hydrophilic Interaction Liquid Chromatography
**HLM**	Human liver microsomes
**HMDB**	Human Metabolome Database
**HPLC**	High performance liquid chromatography
**IC**	Inhibitory concentration
**LC**	Liquid chromatography
**LOAEL**	Lowest observed effect level
**LTRA**	Leukotriene D4 receptor antagonists
**MI**	Mitochondrial damage
**MS**	Mass Spectrometry
**MTBE**	Methyl tert -Butyl ether (MTBE)
**MTs**	3D microtissues
**MUFA**	Monounsaturated fatty acids
**NAD**	Nicotinamide
**NADH**	Nicotinamide adenine dinucleotide
**NMR**	Nuclear Magnetic Resonance
**NPS**	Nanoparticles
**NRU**	Neutral red uptake
**OPLS-DA**	Orthogonal least squares discriminant analysis
**OS**	Oxidative stress
**PCA**	Principal Component Analysis
**PC-DFA**	Principal component discriminant function analysis
**PFOA**	Perfluorooctanoic acid
**PHHs**	Primary Human Hepatocytes
**PI**	Phosphatidylinositol
**PoD**	Point of departure
**QA**	Quality assessment
**QC**	Quality control
**RSD**	Relative standard deviation
**RT**	Retention Time
**SAM**	S-Adenosylmethionine
**SCFA**	Short chain fatty acids
**SM**	Sphingomyelin
**SOPs**	Standard Operational Procedures
**ST**	Steatosis
**SVRC**	Support Vector Regression
**TCC**	Triclocarban
**TCDD**	2,3,7,8-Tetrachlorodibenzo-p-dioxin
**TG**	Triacylglycerol
**TPP**	Triphenyl phosphate
**UGTs**	Uridine 5′-diphospho-glucuronosyltransferases
**UMP**	Uridine monophosphate
**XMP**	Xanthosine monophosphate
**ZZ**	Gardeniae Fructus
**α-PBP**	α-pyrrolidinobutiophenone
**α-PEP**	α-pyrrolidinoheptaphenone
